# *Caenorhabditis elegans* Multi-Tracker Based on a Modified Skeleton Algorithm

**DOI:** 10.3390/s21165622

**Published:** 2021-08-20

**Authors:** Pablo E. Layana Castro, Joan Carles Puchalt, Antonio García Garví, Antonio-José Sánchez-Salmerón

**Affiliations:** Instituto de Automática e Informática Industrial, Universitat Politècnica de València, 46022 Valencia, Spain; pablacas@doctor.upv.es (P.E.L.C.); juapucro@doctor.upv.es (J.C.P.); angar25a@etsid.upv.es (A.G.G.)

**Keywords:** *C. elegans* assays, lifespan, healthspan, image detection, multi-tracker, standard Petri dishes

## Abstract

Automatic tracking of *Caenorhabditis elegans* (*C. egans*) in standard Petri dishes is challenging due to high-resolution image requirements when fully monitoring a Petri dish, but mainly due to potential losses of individual worm identity caused by aggregation of worms, overlaps and body contact. To date, trackers only automate tests for individual worm behaviors, canceling data when body contact occurs. However, essays automating contact behaviors still require solutions to this problem. In this work, we propose a solution to this difficulty using computer vision techniques. On the one hand, a skeletonization method is applied to extract skeletons in overlap and contact situations. On the other hand, new optimization methods are proposed to solve the identity problem during these situations. Experiments were performed with 70 tracks and 3779 poses (skeletons) of *C. elegans*. Several cost functions with different criteria have been evaluated, and the best results gave an accuracy of 99.42% in overlapping with other worms and noise on the plate using the modified skeleton algorithm and 98.73% precision using the classical skeleton algorithm.

## 1. Introduction

The nematode *Caenorhabditis elegans* (*C. elegans*) is a widely studied animal model, as its diverse age-related behavioral patterns provide valuable information on the function of its nervous system and is, therefore, an attractive model to evaluate the effects of mutations [1]. This facilitates the study and treatment of aging, as well as age-related pathologies and neurodegenerative disorders in humans at advanced ages [2,3]. Many of these studies have shown that automatic tracking applications based on computer vision systems help to reduce the manual cost of data acquisition and research hours, improving potential observation of the effects of drug trials [4] and improvements in lifespan or “shelf-life” [5,6,7]. These systems provide quantitative information on alterations in the individual motility and behavior of worms produced by chemical substances in their environment (chemotaxis), providing statistical data that allow further research in the field of health and wellness.

*C. elegans* demonstrate group behavior [8,9,10], among the best known are courtship, mating, aggression, rearing and foraging. Group behavior assays are currently being performed, for example, research into the effect of $0_{2}$ in food search analysis, and aggregation [11,12]. These assays, like others, are visualized manually, due to the complexity of solving the identification problem during an overlapping or body contact of these worms. Currently, the automatic [13,14,15,16,17,18,19,20,21] or semi-automatic applications discard the data from tracks where there are these particular cases (overlapping and bodies contacts) [22,23,24]. Overlapping can take place among worms or may also be due to plate noise. Plate noise was defined as segmentation errors due to edges, or opaque waste in the plate.

Certain applications use diverse techniques and methods such as length [25], smoothness [26,27], previous segmentation [28], or other complex methods [29,30,31,32,33,34] to solve tracking problems during aggregation. The aforementioned methods were tested by our team and results indicated that previous segmentation is one of the most significant criteria to help identify worms within an aggregation. This was taken as a starting point to design different trackers, adding and combining numerous criteria, and it was found that the criteria of completeness and color used with the improvement of the form of skeletonizing [35] can help to identity the worms in the next image. Furthermore, we introduced three new criteria: length, smoothness, and noise, revealing that they can be useful in re-identification.

We present different multi-trackers which, unlike any other trackers, are fully automatic (Table 1). Segmentation and identification of the edge, the interior rim of the Petri dish (area of interest), and segmentation tracks made by worms are performed without the intervention of human operators. These new methods show worm-tracking accuracy of above 98% in nematode aggregations and problems related to plate noise. The best result reaches 99.42% accuracy.

## 2. Materials and Methods

### 2.1. Nematode Strains and Culture

The strains (N2) and CB1370 *daf-2* (e1370) used in the study were provided by the University of Minnesota Caenorhabditis Genetics Center. The *C. elegans* were kept at 20 °C and cultured on 55 mm diameter NGM plates with 1 µg/mL of fungizone to prevent fungal contamination [38]. Escherichia coli (*E. coli*) strain OP50 was used as standard prey for *C. elegans* in the laboratory. FUdR (0.2 mM) was used to prevent replication. The cultured plates were 10, 15, 30, 60 and 100 adult-young worms synchronized from worm eggs incubated at 20 °C. This variety in the number of worms per plate allowed to obtain aggregations of two *C. elegans* or more.

### 2.2. Proposed Tracking Method

At present, automatic or semi-automatic trackers discard the data of tracks where there are overlap or body contacts, due to the difficulty of solving the identity of each individual in these situations. Our method does solve these situations using a fully automated pipeline based on an intelligent active backlight, a modified skeletonization method and new criteria to solve the final optimization problem.

**Figure 1:**
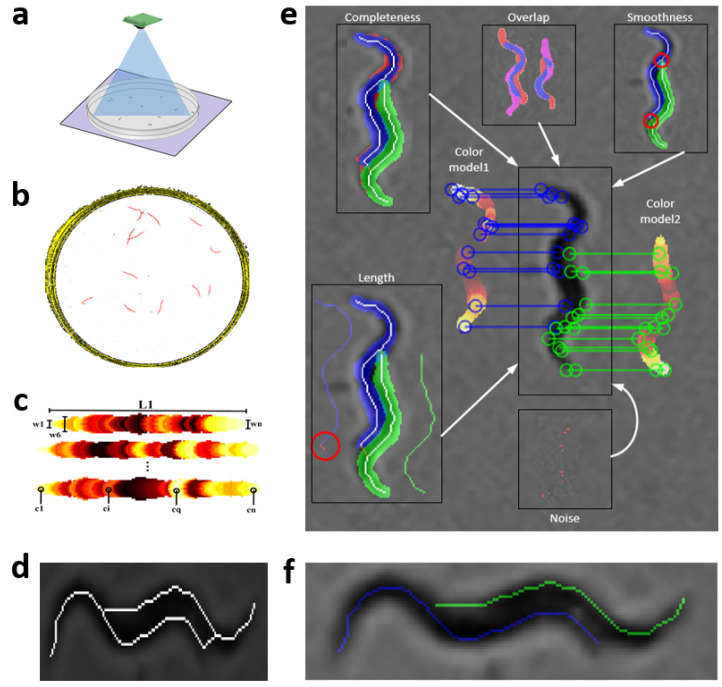
General scheme of image processing. The image shows the different stages that the tracker goes through to obtain the results of the skeletons and to follow all the worms within the plate. (**a**) Image acquisition [37]. (**b**) Pre-processing image. (**c**) Worm models. (**d**) Improved skeleton using proposed method [35]. (**e**) Optimization method. (**f**) Optimization results (predicted poses).

Steps of the proposed tracking method are described in Figure 1a–f, and more detailed in Section 2.3, Section 2.4, Section 2.5, Section 2.6 and Section 2.7, respectively. This method starts with the acquisition of sequences of *C. elegans* images, Figure 1a. These image sequences are then processed (Figure 1b) to obtain mathematical models of *C. elegans* Figure 1c, and possible solutions to these in an aggregation. Finally, the result (Figure 1f) is the optimal minimization cost between models and possible solutions.

### 2.3. Image Acquisition

The image acquisition process was performed using the capture system [37]. To use this system, first, a laboratory operator removed the plates from the incubator, then the plates were analyzed to find condensation on the covers, if so, they were removed, otherwise, the image sequence was captured. The system [37] is automatic and captures an image every second. *Escherichia coli* (*E. coli*) strain OP50 was placed in the center of the plate to capture the worms inside the Petri dish and not scaling the edges or near them. The intelligent active backlighting method was used as the illumination technique [36]. This method is more robust than standard backlighting methods, as it allows us to obtain constant intensity values for the bottom of the Petri dish and the worms (greater than 48 and less than 35, respectively). This facilitated automatic segmentation with fixed thresholds on all images.

The image sequence was acquired at a resolution of 1944 × 1944 pixels and a frequency of 1 Hz (1 image per second) using the system [37] (Table 2).

This image acquisition system is open hardware and its assembly procedure, parts and description are described in detail in another work [37]. Worm tracking, using these image acquisition conditions, is a very challenging problem. An image resolution of 1944 × 1944 pixels is the lowest able to detect worms, when a complete Petri dish (55 mm. of diameter) is monitored with a fixed camera. In addition, this problem was solved by using a low frame rate of 1 Hz. The dataset collected was composed by sequences of 30 images where contact events between worms occurred, to perform all the experiments.

### 2.4. Image Processing

Image processing began with the segmentation of the region of interest and *C. elegans* tracks in the image sequence (white circle and red track in Figure 1b). To obtain the region of interest, first, a segmentation was carried out on all the images of the sequence using a threshold with a fixed intensity value (35 in the gray scale). Then, an AND operation was executed between all the segmented images, the result obtained went through a “Fillhole” operation to fill small holes. The region of interest was selected as the largest connected component of the resulting image. It is important to mention that the use of a fixed threshold for all images is due to the intelligent active backlighting method as a lighting technique [36], as this system allows to conserve background intensity values constantly.

In parallel to the previous step, the *C. elegans* tracks were segmented with a process using different threshold levels Figure 1b. Threshold levels were below 35 on the gray scale. The resulting segmentations went through two filters in order to eliminate those tracks that did not correspond to worms. In the first stage, those tracks with an area smaller than the minimum area of a worm were filtered. The second filter analyzed the skeleton of each image, if the skeleton did not correspond to a minimum expected length, it was classified as noise. The results of the number of skeletons found in each image were stored in a 30 × N matrix, where N is total number of tracks and 30 is the number of images in the sequence.

Due to low resolution of the worms, a scale factor of 3 was applied to increase resolution. This process was applied by obtaining the model to the end of tracking. The model of each worm was obtained by analyzing 30 × N matrix, finding image “k” of 30 images, where worms were further apart from each other, and their ends, head–tail, were also separate. Tracking of each worm started from image k + 1 to image 30 and from image k − 1 to the first. The skeletonization method proposed in previous work [35] was used in each image. This method used distance transformation [39] to obtain possible worm skeletons, and through of an optimization method using different criteria found the best skeleton prediction. Once the tracking process had finished, the results were reconverted to the original scale to be saved.

### 2.5. Worm Model

**Figure 2:**
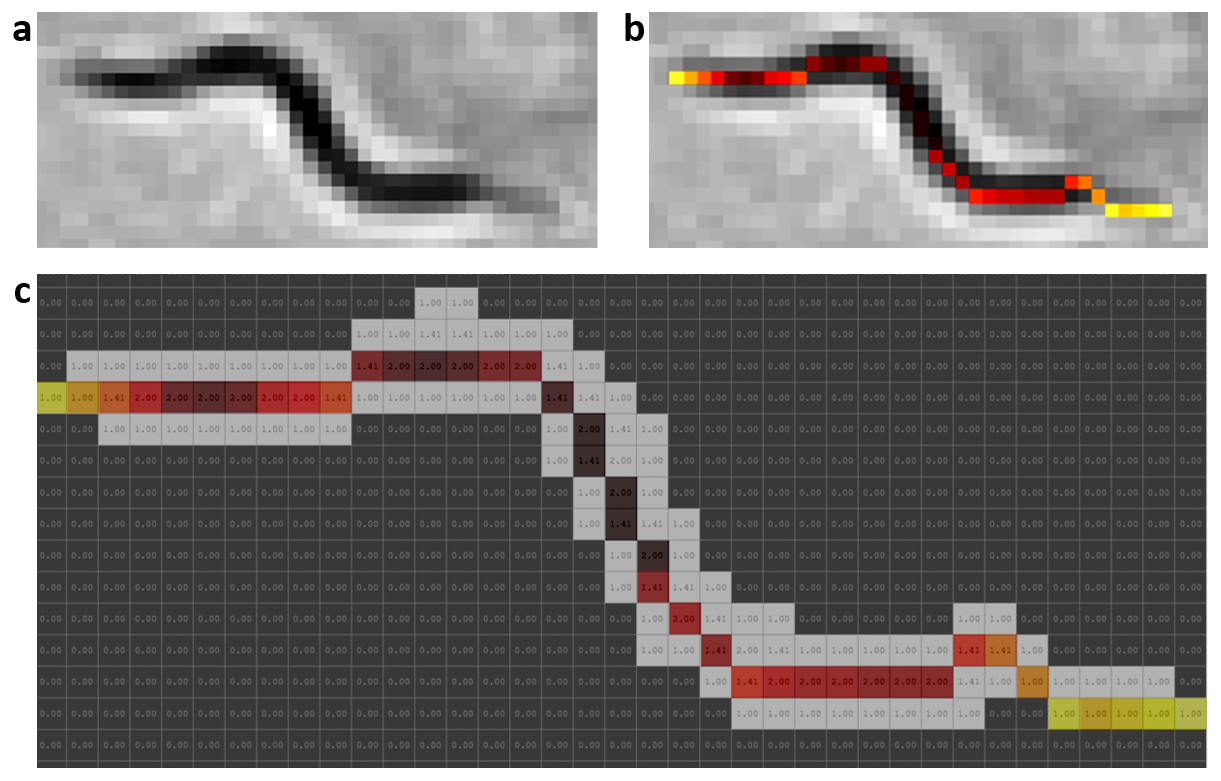
Prediction model. Skeleton gray values were changed with a HOT color map for better visualization. (**a**) Grayscale image. (**b**) Color values obtained from grayscale image. (**c**) Values of widths marked with the colors of the model; the length is the total pixels in the skeleton.

At present, there are different skeletonization methods [39,40]. Matlab’s bwmorph function was used as a classic skeletonization method to obtain worm skeleton model (color pixels in Figure 2b). The proposed worm model consists of width and color values along the skeleton of each individual, Figure 2c. The width values are obtained using classical skeleton in resulting image after using distance transformation function on segmentation of the image “k”. Grayscale image is shown in Figure 2a, while color values of pixels are obtained using the classical skeleton in gray image in Figure 2b. The length value is the total number of pixels in the skeleton. The length model is averaged while the *C. elegans* are separate and tracking progresses.

### 2.6. Extraction of Possible Solutions

**Figure 3:**
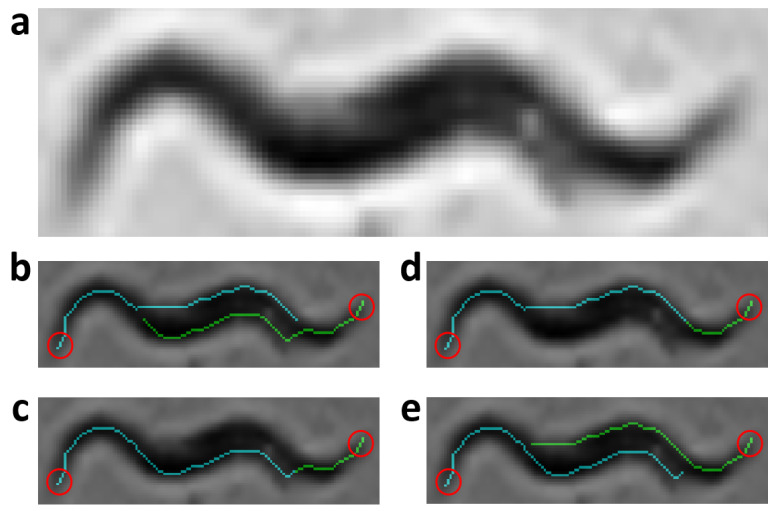
Possible solutions. Circles in red mark the starting point to find the possible skeleton. Cyan and green lines are the possible solutions for each worm. (**a**) Grayscale image. (**b**–**e**) Possible solutions.

The skeletonization method proposed in the previous work [35], unlike classical methods, enables the separation of aggregated worms (Figure 3a), creating new paths in the skeleton, and some possible solutions for each worm (Figure 3b–e). Maximum and minimum values of the width vector are used in the distance to transform images of each segmentation to find this new skeleton as mentioned in [35]. The possible solution skeletons are obtained from a recursive function, which runs through ends and branch points of the new skeleton that overlap the previous segmentation of the worm’s body (red circle in Figure 3b–e).

### 2.7. Optimization Method

The prediction “S” of the following postures will be the possible solution with the minimum value within all the possible “*P*” combinations of skeletons in one segmentation (1). The value of each possible combination “$C_{P}$” of skeletons is obtained from the sum of criteria “m”, for the number of worms “n” in an aggregation (2). The criterion “$C_{p}$” is evaluated for each possible worm “i” and for each criterion “j”. The criteria analyzed were the length of skeleton, overlap with the previous body, completeness, smoothness of the skeleton, noise in segmentation and the colors of each worm.

(1) $$S=\;\underset{p}{arg min}\left(C_{p}\right)$$

(2) $$C_{p}=\sum_{j=1}^{m}\left(\sum_{i=1}^{n}C_{j}^{i}\right)$$

The length and color criteria prevent the prediction differing from the model in length and color. The smoothness criterion prevents sudden changes in the direction of the skeletons. The completeness criterion prevents the current segmentation from being incomplete. The overlap criterion with the previous body prevents the identity change during aggregation. Furthermore, the noise criterion prevents the skeleton prediction falling on the plate noise.

The reconstruction of the body of each worm was used for the evaluation of the different criteria. This was performed by using the skeleton pixels in each possible prediction with the width and color values obtained in the model (prediction start), respectively, for each individual.

#### 2.7.1. Length Criterion

**Figure 4:**
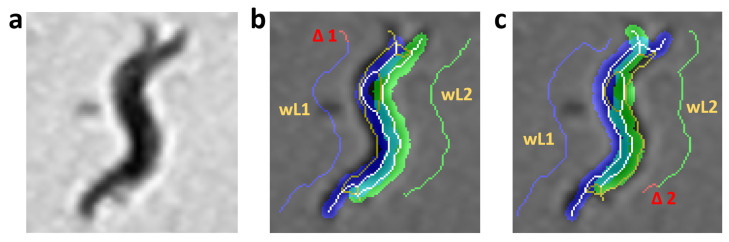
Length criterion evaluation. The pixels in red are the length error. The yellow and white pixels are the resulting skeleton using the improved form of skeletonizing. The white pixels are the pixels of the skeleton prediction, which are used to reconstruct the body of each worm (segmentation in blue and green). (**a**) Grayscale image. (**b**) Length criterion evaluation with length error in worm1 (blue). (**c**) Length criterion evaluation with length error in worm2 (green).

The length criterion “$C_{L}$” is obtained from the sum of the multiplication of average squared width [$\bar{W_{i}^{2}}$] with the difference in length ($\Delta_{i}$), (3). This difference is obtained from subtraction between the model length of each worm ($L_{i}$) and length of the skeleton obtained ($WL_{i}$). The average squared of the width was used so that the resulting length criterion is as significant as the rest of the criteria where the error in pixels was evaluated. Figure 4a shows the aggregation of two worms in a gray scale, and Figure 4b,c shows possible posture predictions where length errors occur.

(3) $$C_{L}=\sum_{i=1}^{n}\left(\Delta_{i}\bar{W_{i}^{2}}\right)$$

#### 2.7.2. Overlap Criterion

**Figure 5:**
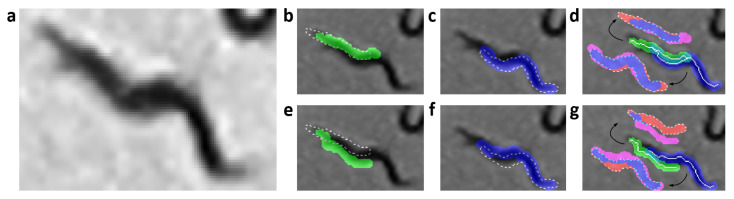
Overlap criterion evaluation. The pixels in red and magenta are the error of overlap with the previous state. The white pixels are the pixels of the skeleton prediction, which are used to reconstruct the body of each worm (segmentation in blue and green). The dashed line segmentation is the previous state segmentation. (**a**) Grayscale image. (**b**,**e**) Previous state ($Bps_{px}$) in white dashed line and current state ($Bcs_{px}$) in green. (**c**,**f**) Previous state ($Bps_{px}$ in white dashed line and current state ($Bcs_{px}$) in blue. (**d**) Evaluation with low overlap criterion. (**g**) Evaluation with high overlap criterion.

The overlap criterion “$C_{O}$” is obtained from the sum of the absolute difference of the reconstruction of the worm’s body in the previous state, $B_{Ppx}$, (white dashed line in Figure 5b,c,e,f) and the current state, $B_{Cpx}$, (green and blue segmentation in Figure 5b,c,e,f) for each possible worm “*i*” in the aggregation (4) and (5). This is done for all “m” pixels in each reconstruction. Figure 5a shows in gray scale the aggregation of two worms, Figure 5d,g shows the next postures predictions (blue and green segmentation), parallel to these in dashed lines showing the previous state ($B_{Ppx}$) and the rest show the current state ($B_{Cpx}$). In Figure 5d it can be seen that the overlap criterion is low because it belongs to the same worms, while in Figure 5g it is higher due to the identity change.

(4) $$C_{O}^{i}=\sum_{px=1}^{m}\left\{\begin{array}{c} 1\;\;if\;\left|B_{Ppx}-B_{Cpx}\right|==1\\ 0 \end{array}\right.$$

(5) $$C_{O}=\sum_{i=1}^{n}C_{O}^{i}$$

#### 2.7.3. Completeness Criterion

**Figure 6:**
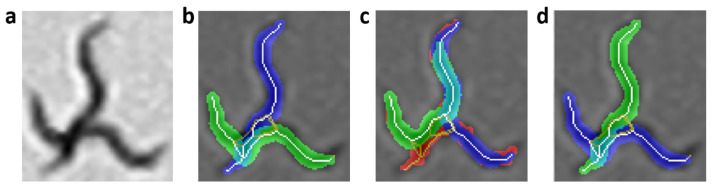
Completeness criterion evaluation. The pixels in red are the completeness error. The yellow and white pixels are the resulting skeleton using the improved form of skeletonizing. The white pixels are the pixels of the skeleton prediction, which are used to reconstruct the body of each worm (segmentation in blue and green). (**a**) Grayscale image. (**b**) Correct prediction with low completeness criterion. (**c**) Incorrect prediction with high completeness criterion. (**d**) Identities changed and with the same completeness criterion as image (**b**).

The completeness criterion “$C_{Cp}$” is obtained from the sum of the absolute difference of the current segmentation of the image ($A_{S}$) and the reconstruction of each possible worm body “*i*” in the current state ($B_{Cpx}$) (6) and (7). This is done for all “*m*” pixels in each reconstruction or current segmentation. Figure 6a shows the aggregation of two worms in gray scale, Figure 6b–d shows completeness values for each image. Figure 6b is the correct prediction, while Figure 6c shows incorrect prediction, due to the identity change and final extremes. Figure 6d shows low completeness criterion and an incorrect prediction too, due to the change of identity in both worms.

(6) $$C_{Cp}^{i}=\sum_{px=1}^{m}\left\{\begin{array}{c} 1\;\;if\;\left|A_{Spx}-B_{Cpx}\right|==1\\ 0 \end{array}\right.$$

(7) $$C_{Cp}=\sum_{i=1}^{n}C_{Cp}^{i}$$

#### 2.7.4. Smoothness Criterion

**Figure 7:**
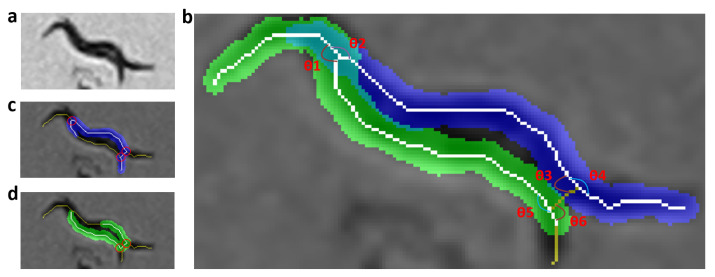
Smoothness criterion evaluation. The yellow and white pixels are the resulting skeleton using the improved skeletonizing method. The white pixels are the pixels of the skeleton prediction, which are used to reconstruct the body of each worm (segmentation in blue and green). (**a**) Grayscale image. (**b**) Evaluation of smoothness, angles in red are those that have more weight and increase the index of smoothness. (**c**) Incorrect prediction of worm1 with high softness criterion. (**d**) Incorrect prediction of worm2 with high softness criterion.

The smoothness criterion “$C_{S}$” is obtained from the average of the absolute values of the angles obtained for the “nk” pixels of the skeleton and for each worm “i” in the segmentation (9). For each pixel of the skeleton, there is an angle ($\theta_{px}$), which is obtained by an average of the sum of “nA” angles before and “nA” angles after divided by the total pixels found “c” (8). The value of “c” will be 2*nA if there are “nA” pixels before and after the pixel to be evaluated (8). Figure 7a shows the aggregation of two worms in gray scale, Figure 7b shows a possible prediction of skeletons with low softness criterion, while Figure 7c,d show possible predictions of skeletons with high softness criterion.

(8) $$\theta_{px}=\frac{\sum_{a=x-nA}^{x+nA}\theta_{a}}{c}$$

(9) $$C_{S}=\sum_{i=1}^{n}\left(\sum_{px=1}^{nk}\frac{|\theta_{px}|}{nk}\right)$$

#### 2.7.5. Noise Criterion

**Figure 8:**
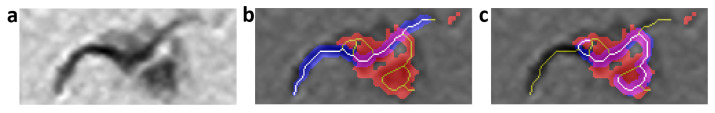
Noise criterion evaluation. The pixels in magenta (intersection of blue and red) are the noise error. The yellow and white pixels are the resulting skeleton using the improved form of skeletonizing. The white pixels are the pixels of the skeleton prediction, which are used to reconstruct the worm’s body (segmentation in blue). (**a**) Grayscale image. (**b**) Correct prediction with low noise criterion. (**c**) Incorrect prediction with high noise criterion.

The noise criterion “$C_{N}$” is obtained from the intersection of the noise segmentation ($N_{S}$) and the reconstruction of each body of a possible worm “i” in the current state ($B_{Cpx}$) (10) and (11). This is done for all “m” pixels in each reconstruction or noise segmentation. Figure 8a shows the aggregation of one worm with noise in a gray scale, while Figure 8b shows the correct prediction (segmentation in blue) with a low noise criterion (segmentation in magenta). Figure 8c shows an incorrect prediction (blue-magenta segmentation) with a high noise index (magenta segmentation).

(10) $$C_{N}^{i}=\sum_{px=1}^{m}\left\{\begin{array}{c} 1\;\;if\;\left|N_{Spx}\&B_{Cpx}\right|==1\\ 0 \end{array}\right.$$

(11) $$C_{N}=\sum_{i=1}^{n}C_{N}^{i}$$

#### 2.7.6. Color Criterion

**Figure 9:**
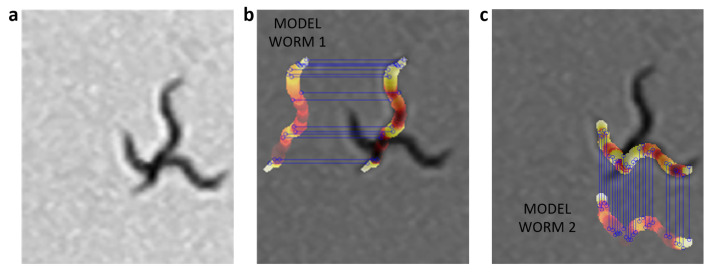
Color criterion evaluation. For each possible skeleton, its color values are obtained using the current grayscale image, and with those color values and the width values of the model, each worm is reconstructed and compared with the reconstruction of the model (model worm). The gray values were changed for a HOT color map in order to better visualize them. The blue lines indicate those pixels different from the model. (**a**) Grayscale image. (**b**) Comparison of worm1 model with current prediction of worm1. (**c**) Comparison of worm2 model with current prediction of worm2.

The color criterion “$C_{Cl}$” is obtained by adding all the pixels with an absolute difference greater than the threshold value (U = 1) of the color model ($C_{M}$) with respect to the reconstruction of the current body ($C_{A}$) (12) for each worm in the aggregation (13). This means that the error only increases the value by 1 if the absolute difference between a pixel of the reconstruction of the body of the possible worm “i” using the color model ($C_{Mpx}$) and the reconstruction of the same worm “i” in the current segmentation ($C_{Apx}$) is greater than the threshold value. This is done for all “m” pixels in the reconstruction. For both worm body reconstructions (model and current prediction), the same width and length values are used, in order to compare pixels 1 to 1. Figure 9a shows the aggregation of two worms in gray scale, while Figure 9b,c shows the comparison of each prediction with its respective model.

(12) $$C_{Cl}^{i}=\sum_{px=1}^{m}\left\{\begin{array}{c} 1\;\;if\;\left|C_{Mpx}-C_{Apx}\right|>U\\ 0 \end{array}\right.$$

(13) $$C_{Cl}=\sum_{i=1}^{n}C_{Cl}^{i}$$

### 2.8. Evaluation Method

Manually labeled skeletons were used as a reference to compare all the results. These skeletons were obtained using an application designed to select each pixel belonging to the skeleton of each worm one by one in the image sequence. This operation was performed for all 3779 postures of the 70 plates used. The shape of these nematodes was recovered using a disk-shaped dilation operation of radius equal to half the width (approx. 2 pixels) on the skeletons obtained.

The Jaccard coefficient, or intersection over the union (IoU), was used to measure the degree of precision in locating worms (14). As its name indicates, it is obtained by dividing the total area of the intersection by the union of the elements [41]. For the evaluation, the area of the reconstructed bodies of the manually labeled skeletons was used, skeletons using the skeletonization method proposed in [35] and the classical skeletonization method using the Matlab bwmorph command.

(14) $$IoU=\frac{\sum_{}^{}P_{w1}⋂P_{w2}}{\sum_{}^{}P_{w1}⋃P_{w2}}$$

**Figure 10:**
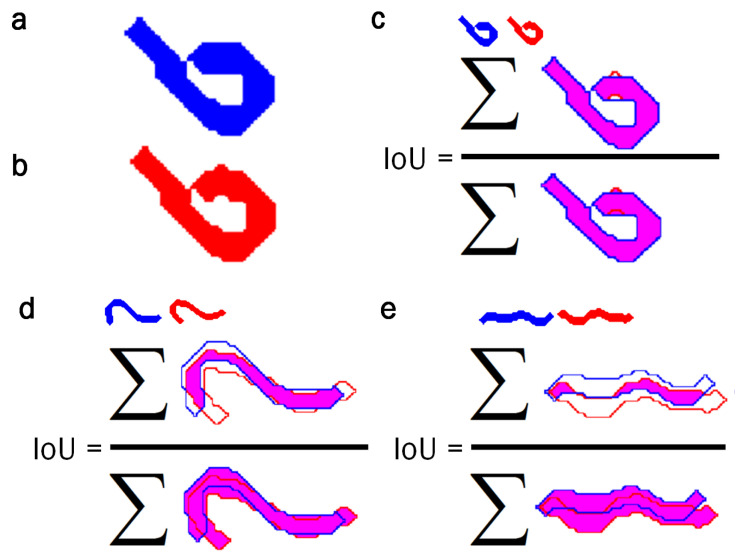
IoU index. This index evaluates how close the response of the automatic method comes to the reference, and compares both results to determine the improvement of one method over the other. The higher the IoU value, the closer the response comes to the reference. The evaluation is performed by reconstructing the skeletons obtained with a radio 2 disk. (**a**) Reconstructed body of the manually labeled skeleton. (**b**) Reconstructed body of the skeleton obtained using the new skeletonization method [35] or the classical method. (**c**–**e**) Evaluation of the reconstructed skeletons.

The IoU index was expected to be higher because a predicted pose (Figure 10b) is compared to an annotated ground-true pose (Figure 10a), which must overlap (Figure 10c–e). The results for the example below are IoU = 0.9784, 0.5667, and 0.2649, respectively.

**Figure A1:**
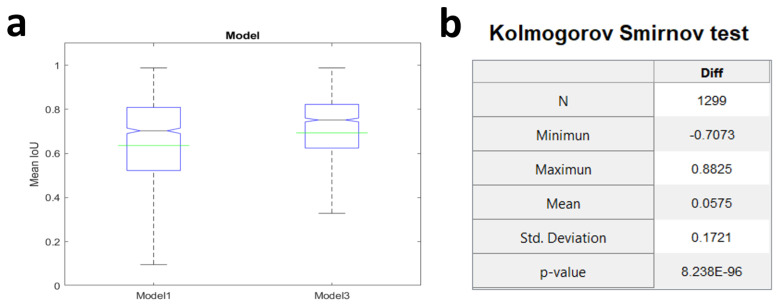
Box plot and normality test of the difference of both models. (**a**) Box plot, green line indicates the mean in both graphs, and gray line indicates the median. Model1, N = 1299, mean = 0.6358, median = 0.7027, std. deviation = 0.2289, variance = 0.0540. Model3, N = 1299, mean = 0.6933, median = 0.7517, std. deviation = 0.1940, variance = 0.0376. (**b**) Normality test on the difference of methods (Model 3–Model 1). The *p*-value obtained was 8.24E-96 less than the significance value of 0.05, so the null hypothesis was rejected and the alternative hypothesis H1 was accepted (data did not come from normal distribution). Once the alternative hypothesis was accepted, Wilcoxon signed ranks test was used to evaluate both methods.

**Figure A2:**
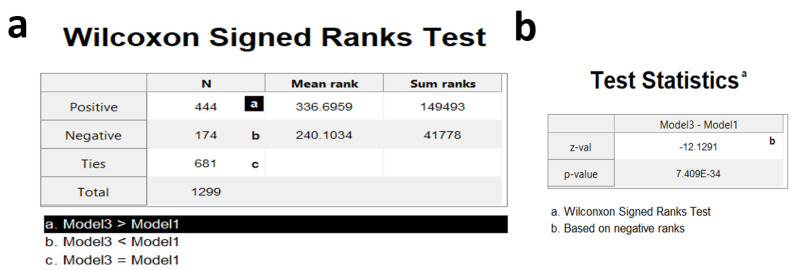
Wilcoxon signed rank test. (**a**) The Wilcoxon signed rank test table shows the difference that exists in 2 related samples through positive, negative and tie ranges. (**b**) *p*-value obtained with Wilcoxon rank test was 741E-34 less than the significance value of 0.05, so it was concluded that there was a statistically significant difference between both models.

**Figure A3:**
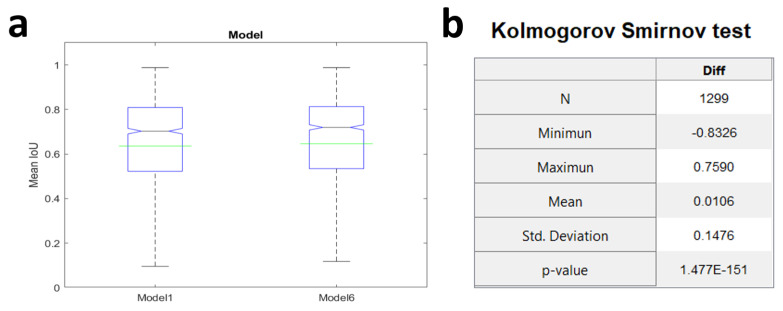
Box plot and normality test of the difference of both models. (**a**) Box plot, green line indicates the mean in both graphs, and gray line indicates the median.Model1, N = 1299, mean = 0.6358, median = 0.7027, std. deviation = 0.2289, variance = 0.0540. Model6, N = 1299, mean = 0.6464, median = 0.7197, std. deviation = 0.2300, variance = 0.0529. (**b**) Normality test on the difference of methods (Model 6–Model 1). The *p*-value obtained was 1.48E-151 less than the significance value of 0.05, so the null hypothesis was rejected and the alternative hypothesis H1 was accepted (data did not come from normal distribution). Once the alternative hypothesis was accepted, Wilcoxon signed ranks test was used to evaluate both methods.

**Figure A4:**
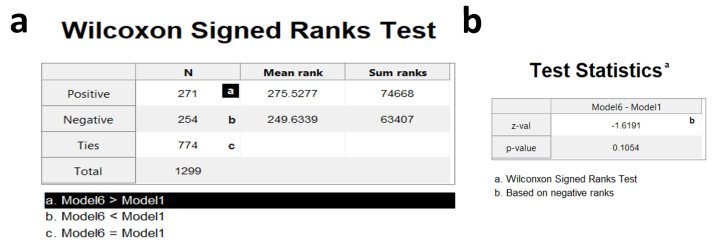
Wilcoxon signed rank test. (**a**) The Wilcoxon signed rank test table shows the difference that exists in two related samples through positive, negative and tie ranges. (**b**) *p*-value obtained with Wilcoxon rank test was 0.1054 less than the significance value of 0.05, so it was concluded that there was not a statistically significant difference between both models.

**Figure A5:**
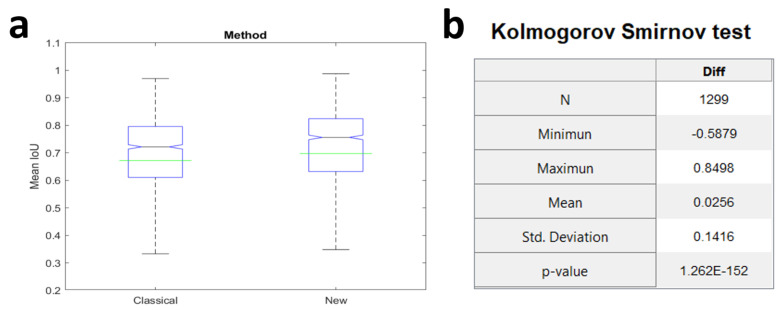
Box plot and normality test of the difference of both methods. (**a**) Box plot, green line indicates the mean in both graphs, and gray line indicates the median. Classical method, N = 1299, mean = 0.6719, median = 0.7218, std. deviation = 0.1842, variance = 0.0339. New method, N = 1299, mean = 0.6975, median = 0.7562, std. deviation = 0.1929, variance = 0.0372. (**b**) Normality test on the difference of methods (New-classical). The *p*-value obtained was 1.26E-152 less than the significance value of 0.05, so the null hypothesis was rejected and the alternative hypothesis H1 was accepted (data did not come from normal distribution). Once the alternative hypothesis was accepted, Wilcoxon signed ranks test was used to evaluate both methods.

**Figure A6:**
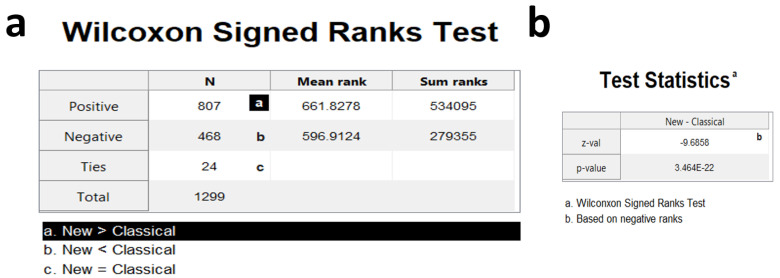
Wilcoxon signed rank test. (**a**) The Wilcoxon signed rank test table shows the difference that exists in two related samples through positive, negative and tie ranges. (**b**) *p*-value obtained with Wilcoxon rank test was 3.46E-22 less than the significance value of 0.05, so it was concluded that there was a statistically significant difference between both methods.

Matlab 2018b Machine Learning Toolbox was used to obtain the comparison statistics between prediction models (Appendix A Figure A1a,b, Figure A2a,b, Figure A3a,b and Figure A4a,b) and the two skeletonization methods (Appendix A Figure A5a,b and Figure A6a,b). The Kolmogorov–Smirnov normality test was used for large samples (n > 50) and the Wilcoxon Signed Ranks test.

## 3. Results

Experiments were performed with 70 plates. Of these, 54 corresponded to plates with 10 and 15 worms, one plate with 30 worms, four plates with 60 worms, and 11 plates with 100 worms, totaling 65,400 worm poses. All these data were analyzed to obtain contact between worms and noise. As demonstrated in [5,14], a higher number of worms per plate will increase the likelihood of contact between them. Nematodes studied were young-adult wild-type (N2) and CB1370, *daf-2* (e1370), as mentioned above.

53 tracks with 3240 poses were used to evaluate aggregation between worms, and 17 tracks with 509 poses for aggregation between worms and noise. The IoU index was used to evaluate the percentage of success in tracking the worms and also to compare both skeletonization methods. The area of worm bodies reconstructed from skeletons obtained manually and skeletons obtained with the two skeletonization methods (new and classical) was used to evaluate the IoU index.

**Figure 11:**
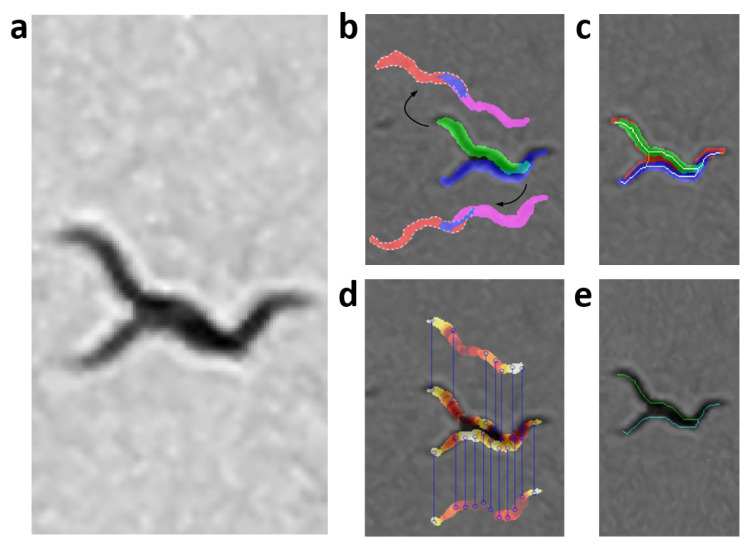
Model 7 evaluation. The yellow and white pixels are the resulting skeleton using the improved form of skeletonizing. The white pixels are the pixels of the skeleton prediction, which are used to reconstruct the body of each worm (segmentation in blue and green). (**a**) Grayscale image. (**b**) Overlap criterion evaluation. (**c**) Completeness criterion evaluation. (**d**) Color criterion evaluation. (**e**) Optimization result.

**Figure A7:**
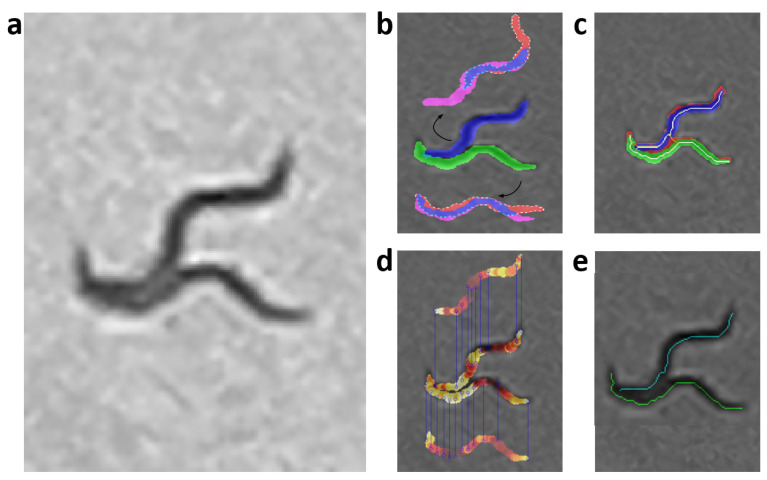
Model 7 evaluation, example 1. The yellow and white pixels are the resulting skeleton using the improved form of skeletonizing. The white pixels are the pixels of the skeleton prediction, which are used to reconstruct the body of each worm (segmentation in blue and green). (**a**) Grayscale image. (**b**) Overlap criterion evaluation. (**c**) Completeness criterion evaluation. (**d**) Color criterion evaluation. (**e**) Optimization result.

**Figure A8:**
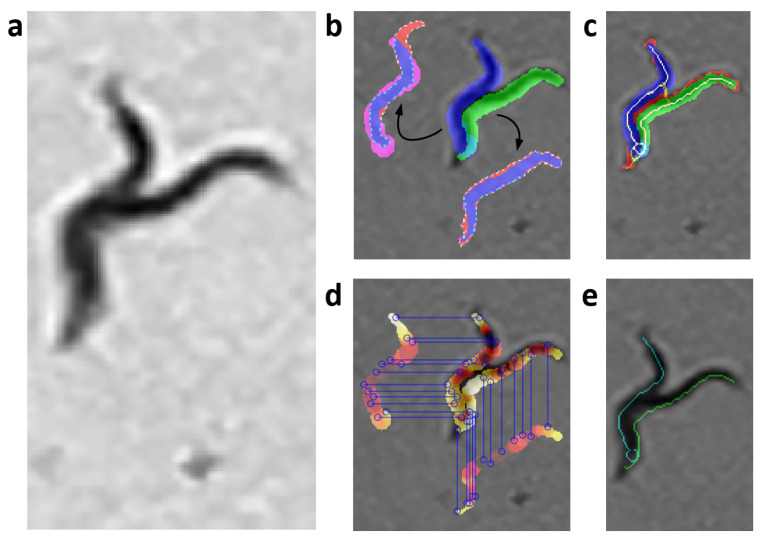
Model 7 evaluation, example 2. The yellow and white pixels are the resulting skeleton using the improved form of skeletonizing. The white pixels are the pixels of the skeleton prediction, which are used to reconstruct the body of each worm (segmentation in blue and green). (**a**) Grayscale image. (**b**) Overlap criterion evaluation. (**c**) Completeness criterion evaluation. (**d**) Color criterion evaluation. (**e**) Optimization result.

**Figure A9:**
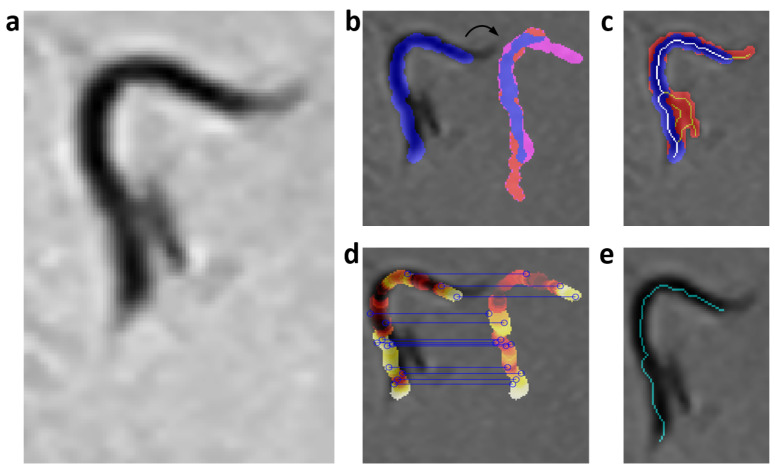
Model 7 evaluation, example 3. The yellow and white pixels are the resulting skeleton using the improved form of skeletonizing. The white pixels are the pixels of the skeleton prediction, which are used to reconstruct the body of each worm (segmentation in blue and green). (**a**) Grayscale image. (**b**) Overlap criterion evaluation. (**c**) Completeness criterion evaluation. (**d**) Color criterion evaluation. (**e**) Optimization result.

**Figure A10:**
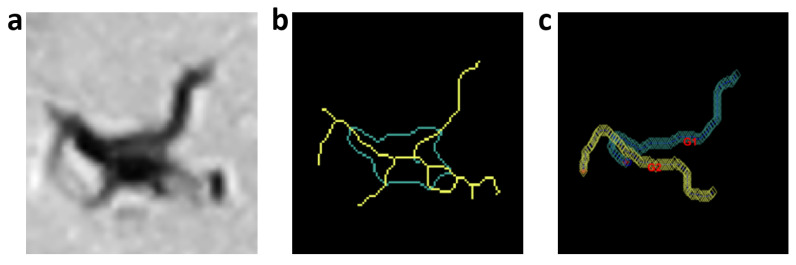
Model 7 evaluation, example 4. Errors occurred by the presence of noise. (**a**) Grayscale image. (**b**) Resulting skeleton using the improved form of skeletonizing. (**c**) Optimization result.

**Figure A11:**
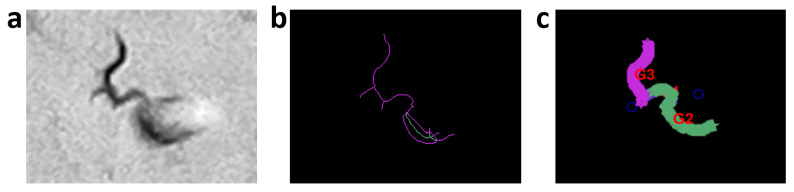
Model 7 evaluation, example 5. Errors occurred by the presence of noise. (**a**) Grayscale image. (**b**) Resulting skeleton using the improved form of skeletonizing (**c**) Optimization result.

Different prediction models were implemented in order to find the most significant criteria. The name of each model has been coded using letters from the criteria names. “O” for overlap, “L” for length, “Cp” for completeness, “N” for noise, “S” for smoothness, and “Cl” for color. The model with the best results was model 7 (OCpCl) with a 99.42% percentage accuracy in aggregated worm tracks and an IoU value of 0.70 in average. Figure 11a–e, shows an example using the model 7, in this image you can see the evaluation of the three criteria of this model and optimization result. Some examples of aggregation cases are presented at the end of the Appendix B using model 7 (Figure A7, Figure A8, Figure A9, Figure A10 and Figure A11).

To measure the percentage accuracy in tracking the worms, non-zero IoU values were used, from the beginning to the end of the tracks. The accuracy of the results of the different prediction models using the 2 skeletonization methods are shown in Table 3. In addition, the average IoU value was obtained for all the prediction models (see Table 4), from the beginning of the aggregation to the end. Then, 790 poses were used in aggregations between worms and 509 poses for worms aggregated with noise, 1299 poses in total.

### Comparison with Other Trackers

At present, automatic or semi-automatic trackers discard the data of tracks where there are overlap or body contacts, due to the difficulty of solving the identity of each individual in these situations. Our method does solve these situations, so a direct comparison cannot be made with the results of other trackers.

**Figure 12:**
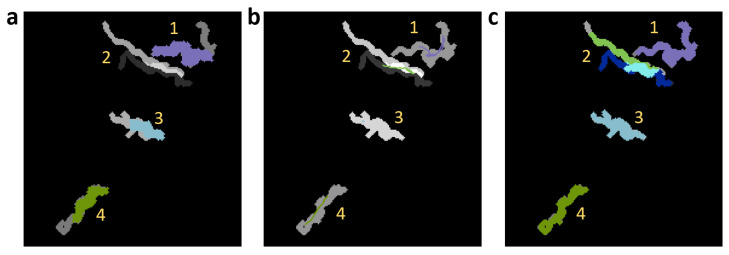
Comparison of trackers. Comparison of reconstruction of *C. elegans* bodies between labeled data (shaded in grays) and predictions obtained with different trackers (shaded in colors). (**a**) Results obtained using tierpsy-tracker [22]. (**b**) Labeled data (grays) compared with colored lines that connect centroids obtained using WF-NTP.v3 [23]. (**c**) Results obtained using model 7.

To compare our method graphically with other trackers (tierpsy-tracker [22], WF-NTP.v3 [23]), labeled data was first shaded in different grays and overlapped predicted data in colors (Figure 12a–c). Errors for each comparison are shown in grayscale. The comparison was made with bodies reconstructed from skeletons (skeleton dilation with disk equal to 2), except for WF-NTP.v3 [23], whose results are skeleton centroid points. The tierpsy-tracker [22] multi-tracker, Figure 12a, did not resolve path 2 where aggregation occurs, while the other tracks (1, 3, 4) were partially resolved, due to the occlusions that the worms make on themselves. The multi-tacker WF-NTP.v3 [23], Figure 12b, did not solve the identity problem in track 2. Furthermore, like the previous multi-tracker it presented problems in the tracks with occlusions (1, 3, 4). Model 7, on the other hand, had almost zero errors, tracked all worms, resolved the identity in the aggregation of track 2 and the occlusions in the remaining tracks, Figure 12c.

## 4. Discussion

*Caenorhabditis elegans* are aggregated in different ways, such as aggregation of end parts (head or tail), partial aggregation of bodies, and aggregation of parallel bodies, among others. The experiments were conducted with the above mentioned methods with a single criterion and found that the overlapping criterion with the previous prediction is the most significant. This criterion allows part of the previous state to be preserved, helping to solve the next state. A result of 98.49% was obtained using this criterion individually. However, when aggregation gives rise to an overlap in most worms, it is difficult to identify them, even for human observers. To solve this problem, different tests were designed, combining the criteria mentioned in the optimization methods section.

The completeness in cases of aggregation, where there are overlaps between aggregated bodies, partial and total identity changes could be obtained, as observed in the optimization method Figure 6c,d. When using the overlap criterion with completeness criterion, a percentage accuracy of 99.13% was achieved. As shown in Appendix A (Figure A1a,b and Figure A2a,b) this criterion is statistically significant, helping to solve some cases where the overlap criterion presented problems, Figure 5b.

The color criterion also helped to improve worm prediction, although not so significant (Appendix A Figure A3a,b and Figure A4a,b). The end portion of *C. elegan* (tail) has a greater number of light pixels (higher gray levels) than the head, where there are fewer. These features are important because before, during and after an aggregation one of the final parts remains visible, and with this color singularity a more accurate prediction can be obtained, helping to solve the identity problem after an aggregation between worms or noise. The use of these three criteria (overlap, completeness, and color) allowed us to obtain a percentage accuracy of 99.42%.

The length, smoothness, and noise criteria were discarded because when they were used individually or together with the overlap criterion, their percentage accuracy decreased. On the one hand, the problem with the smoothness criterion was due to the low resolution of the worms in our images, which provided a poor estimate of this criterion. On the other hand, the problem with the length criterion was due to errors in the length model and the increase or decrease in the length of worms owing to overlaps. The problem with the noise criterion was due to, when the worm is visualized against background noise, many possible solutions were generated.

Finally, it is worth mentioning that the best combination of criteria depends on image quality. In this work, we demonstrated that the combination of the three criteria mentioned above (overlap, completeness, and color) was the best option for automatic tracking of interaction behaviors among *C. elegans* (contacts or overlapping) with our low-resolution dataset.

## 5. Conclusions

This paper presents a method for tracking multiple *C. elegans* in standard Petri dishes where some worms can come into contact or overlap. This method was evaluated in a difficult scenario using a low-image resolution and a low frame rate. Using an optimizer with the appropriate criteria (overlap, completeness, and color) was shown to solve many worm overlap and contact situations. The accuracy obtained under these conditions was 99.42% and 98.73% using the modified skeleton algorithm and the classical skeleton algorithm, respectively. In addition, the proposed method employs an improved active backlight system and an improved skeletonization algorithm. The active backlight system provides fixed gray levels in all captured images, which allows automatic segmentation using a fixed threshold. The improved skeletonization algorithm uses width information from each worm model to extract skeletons, enabling the tracking of worms moving in parallel (side by side). Our proposal, unlike other trackers that discard worm overlaps and contacts, solves many of these situations, increasing the number of worms tracked in a Petri dish and therefore paving the way to automate new assays where interaction between worms occurs.

## Figures and Tables

**Table 1:** Comparison with other multi-trackers. This table shows the comparison of our multi-tracker with respect to others (tierpsy-tracker [22], WF-NTP.v3 [23]).

Comparative Table				
**Name**	**Illumination Technique**	**Features**	**Method**	**Situations solved**
Tierpsy Tracker [22]	Standard backlight	Manual parameter setting	Skeletons, outlines and segmentations	Individual tracking
WF-NTP.v3 [23]	Flat-field illumination	Manual parameter setting	Skeletons and centroids	Individual tracking
Ours	Active backlight system [36,37]	Fully automatic	Improved skeleton and segmentations	Individual tracking, overlaps, body contacts, rolled worms and occlusions.

**Table 2:** Components of the data acquisition system.

System Components		
N °	Name	Description
1	Raspberry Pi V3 b+	Procesador: 64-bit ARM Cortex-A53, 1.4 GHz RAM Size: 1 GB LPDDR2 SDRAM
2	Raspberry Pi Camera V1.3	Sensor: OmniVision OV5647 Pixel resolution: 2592 × 1944 Pixel size: 1.4 × 1.4 µm Field of view: 53.50° × 41.41° Optical size: 1/4′′ Focal length: 2.9
3	Raspberry Pi display	Screen display size: 7′′ Resolution: 800 × 480 and 60 fps Color: 24-bit RGB colour

**Table 3:** Comparative table of percentage accuracy of postures for models and methods. The table shows the percentage accuracy of poses (skeletons) for each model and method used during the tracking of *C. elegans*. Overall, 3240 poses were used to evaluate tracks where there is aggregation of two or more worms, and 509 poses to evaluate the aggregation between worms and noise on the plate.

Worms Aggregation			
**N${}^{\circ }$**	**Model**	**New**	**Classical**
1	O	98.49	97.80
2	OL	98.15	97.99
3	OCp	99.13	98.09
4	ON	98.09	97.88
5	OS	98.07	97.72
6	OCl	98.57	98.20
7	OCpCl	**99.42**	**98.73**

**Table 4:** Summary of model and method comparison. This table shows the results for each prediction model and two skeletonization methods, new [35] and classical (using the Matlab bwmorph function). The results column indicates the percentage value of the improvement using the new skeleton with respect to the classic skeletonization.

			Average IoU		Standard Deviation		Results %
**N${}^{\circ }$**	**Model**	**Total Pose**	**New**	**Classical**	**New**	**Classical**	**Improvement**
1	O	1299	0.64	0.63	0.23	0.22	0.39
2	OL	1299	0.63	0.63	0.23	0.22	0.10
3	OCp	1299	0.69	**0.67**	0.19	0.19	2.61
4	ON	1299	0.63	0.63	0.24	0.22	0.50
5	OS	1299	0.63	0.63	0.23	0.22	0.04
6	OCl	1299	0.65	0.65	0.23	0.20	−0.15
7	OCpCl	1299	**0.70**	**0.67**	0.19	0.18	**2.66**

## Data Availability

The program was developed in Matlab2018b with Windows 10 and works correctly in later versions with Image Processing, Communications and Bioinformatics toolbox. Its source code is in GitHub. It is open-source MIT (Massachusetts Institute of Technology) and can be downloaded from the repository at https://github.com/playanaC/WT_ISA, accessed on 17 August 2021. The dataset with all aggregation experiments can be downloaded from https://active-vision.ai2.upv.es/wp-content/uploads/2021/02/dataset_skeletons.zip, accessed on 17 August 2021.
